# The Effect of Infant Gastric Digestion on Human Maternal Milk Cells

**DOI:** 10.1002/mnfr.202200090

**Published:** 2022-08-31

**Authors:** Rose Doerfler, Jilian R. Melamed, Kathryn A. Whitehead

**Affiliations:** ^1^ Department of Chemical Engineering Carnegie Mellon University Pittsburgh PA 15213 USA; ^2^ Department of Biomedical Engineering Carnegie Mellon University Pittsburgh PA 15213 USA

**Keywords:** human milk, infant digestion, milk cells

## Abstract

**Scope:**

Human breast milk contains a variety of cell types that have potential roles in infant immunity and development. One challenge associates with defining the purpose(s) of milk cells in the infant is a poor understanding of the effect of digestion on cell fate.

**Methods and results:**

This study first demonstrates that milk cell death occurs after gastric digestion in mice. Then flow cytometry and RT‐PCR are used to understand the mechanism of human milk cell death and quantify live cell types before and after simulated gastric digestion. This study finds that digestion in simulated gastric fluid for 30 min reduces cell viability from 72% to 27%, with most cell death is caused by the acidic pH. The primary mechanism of cell death is caspase‐mediated apoptosis. The non‐cellular components of milk offer only mild protection against cell death from stomach acid.

**Conclusions:**

Gastric digestion does not select for a specific resilient cell population to survive—most cell types die in equal proportions in the gastric environment. Taken together, these results provide a foundation with which to understand the fate of human breast milk cells in the infant's intestine and beyond.

## Graphical abstract

Human milk contains maternal cells with potential roles in infant development. This study demonstrates that the majority of cells from human milk die by apoptosis after 30 min of digestion in simulated gastric fluid, without selecting for a resilient cell type. These results provide a foundation to understand the fate of milk cells in the intestine and beyond.

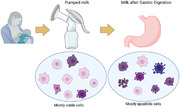

## 1 Introduction

Human breast milk is steadily gaining interest within the scientific community for not only its nutritional components, but also for its diverse populations of maternal cells, extracellular vesicles, and nucleic acids. Part of this interest derives from the remarkable bioactive properties of these components.^[^ ^1^, ^2^, ^3^ ^]^ Human milk contains immune cells, several types of mammary epithelial cells, and stem and progenitor cells reported to differentiate into all three germ layers.^[^ ^4^, ^5^, ^6^ ^]^ The exact composition of milk cell types are dynamic, changing over time in response to mother and infant variables.^[^ ^7^, ^8^ ^]^ Surprisingly, some reports have demonstrated that milk‐derived cells survive the harsh environment of the digestive tract, interact with the infant's mucosal immune system in the intestine, and distribute to organs outside of the digestive system.^[^ ^9^, ^10^ ^]^ These findings are unexpected because the gastrointestinal tract digests most proteins, lipids, and other macromolecules. Studies in multiple animal models including rodents, sheep, and non‐human primates reportedly identified milk‐derived immune cells in the Peyer's patches of the intestine as well as in the lymph nodes, spleen, and liver.^[^ ^11^, ^12^, ^13^ ^]^ More recent reports shifted their focus to milk stem cells, with one study identifying differentiated stem cells in the brains of neonatal mice.^[^ ^14^, ^15^ ^]^ Separate studies confirmed that milk‐derived exosomes survive digestion with their RNA and protein cargo intact.^[^ ^16^, ^17^ ^]^

Although these studies were foundational in establishing that the cells in milk have interesting bioactive properties, many were conducted in the 1980s and 1990s, when it was more difficult to characterize the cell types in milk. Stem‐like milk cells were not described until 2007,^[^ ^18^ ^]^ and many of these initial studies assumed that all milk cells were leukocytes. These limitations have made it difficult to interpret the existing body of research. Using the tools available today, we have the ability to develop a more thorough understanding of milk cell types and their fate. Additionally, no one has identified a mechanism by which these cells survive the infant's digestive system. Thus, it is unclear what fraction of milk‐derived cells survive the infant gastric digestion, and whether some cell types are more vulnerable to digestion than others. To determine the role(s) that human milk cells play in infant development, we must first understand the fate of human milk cells in the infant digestive system.

Herein, we address this knowledge gap by subjecting human milk cells to gastric digestive conditions and analyzing their responses. While the infant gastric environment is substantially milder than in adults, we nonetheless hypothesized that most ingested cells die in the stomach and only robust populations reach the intestine. Our results support this hypothesis—we found that less than 30% of milk cells survive gastric digestion, but the cells detected in the intestinal epithelium were viable. Further, we show the effect of digestion on distinct cell types, the mechanism by which milk cells die, and the protection from the harsh stomach environment offered by the non‐cellular components of milk. Together, these foundational data will support scientists in gaining a better understanding of the role of maternal cells in the infant's body.

## 2 Experimental Section

### 2.1 Animal Experiments

Animal protocols were approved by the institutional animal care and use committee at Carnegie Mellon University (Pittsburgh, PA, USA). All animal experiments were conducted in accordance with Protocol PROTO201800009. Mice were housed under controlled temperature (25 °C) in 12‐hour light‐dark cycles. Animals were given ad libitum access to standard diet and water. C57BL/6J mouse pups at postnatal day 14 were used for all mouse experiments.

### 2.2 In Vivo Milk Cell Viability

Mouse pups at postnatal day 14 were euthanized and the stomach was isolated. A small incision was made in the stomach and the milk bolus was extracted with curved forceps. The milk bolus, made up of soft solid curds, was filtered through a 100 µm cell strainer and smashed with the plunger of a 3 mL syringe. Cells were washed twice with phosphate‐buffered saline (PBS) by centrifugation at 500 × *g*, 4 °C, to remove milk fat and proteins,^[^ ^13^ ^]^ stained with calcein AM violet (Thermo Fisher Scientific), and analyzed using a NovoCyte 3000 flow cytometer (Agilent) to assess cell viability.

### 2.3 Mouse Organ Processing and Flow Cytometry

To track milk‐derived cells through the digestive system, labeled cells from human milk were delivered to mouse pups at postnatal day 14. First, cells were isolated from fresh human milk.^[^ ^19^ ^]^ Milk was combined with PBS in a 1:1 volume ratio and centrifuged for 20 min at 800 × *g* and 4 °C. After the supernatant was removed, the cell pellet was transferred to a clean tube and washed twice with PBS by centrifugation at 600 × *g*, 4 °C, for 7 min each. The resulting human milk cells were stained with CellTracker Deep Red (Thermo Fisher Scientific) according to the manufacturer's protocol, then centrifuged once as done in previous step to remove excess dye. Cells were re‐suspended in PBS and delivered to mice in a bolus of 130 µL containing 10^6^ cells via oral gavage using a flexible polypropylene feeding tube (22ga × 25 mm, Instech Labs). A mouse at postnatal day 14 gavaged with PBS was used as a negative control. Twenty‐four hours later, mice were sacrificed by CO_2_ asphyxiation followed by cervical dislocation, and the intestines and stomach were collected for flow cytometry analysis.

The gastric contents were passed through a cell strainer, centrifuged the resulting cell suspension as described above, and washed with PBS to remove excess proteins and fats. Cells were stained for viability using Calcein‐AM violet, which was a cell‐permeant dye that reacted with intracellular esterases in live cells to produce a fluorescent signal.

Intestines were processed as described previously.^[^ ^20^ ^]^ Briefly, intestines were flushed with PBS, cut longitudinally, and minced into small pieces with scissors. The intestinal epithelium and lamina propria were analyzed as separate samples. To collect the epithelium, intestinal pieces were incubated in 30 mL Roswell Park Memorial Institute medium (RPMI) containing 500 µL fetal bovine serum (FBS) (VWR), 60 µL 0.5 M ethylenediaminetetraacetic acid (EDTA) (Sigma), and 93 µL 5% w/v dithiothreitol DTT) (VWR) at 37 °C for 15 min with vigorous shaking on an Innova 40 orbital incubator‐shaker (New Brunswick Scientific). The EDTA and DTT dissociated the epithelial cells from the intestinal pieces without enzymatic digest, and the epithelial cells were then collected by filtering the intestinal pieces and media through a 70 µm nylon cell strainer (VWR). The epithelial cells passed through the strainer and were pelleted by centrifugation for 10 min at 500 × *g*, 4 °C, and the remaining intestinal pieces were considered to be the lamina propria. These lamina propria pieces were incubated in 25 mL RPMI containing 300 µL FBS, 20 mg collagenase II (activity: ≥125 U mg^−1^), and 10 mg dispase II (activity: ≥0.50 U mg^−1^) (Sigma) at 37 °C for 30 min with gentle shaking on an Innova 40 orbital incubator‐shaker to digest the tissue into a single cell suspension. (Note: the protocol referenced was written to analyze the intestinal cells of adult mice. Since this study analyzing intestines of 14‐day old mouse pups, lower concentrations of enzymes were used.) After centrifugation at 500 × *g*, 4 °C, for 10 min, cells were resuspended in 10 mL RPMI for flow cytometry analysis. Samples were counterstained with propidium iodide (PI) and subsequently analyzed for live, CellTracker Red+ cells using a NovoCyte 3000 (Agilent).

### 2.4 Human Milk Donation

Due to restrictions on human subjects research during the COVD‐19 pandemic, the study chose to use milk from a single donor, who donated milk on multiple days over a period of several months. Milk composition depended on a wide variety of factors, including lactation stage, time of day, maternal BMI, diet, health status, infant gestational age at birth, and environmental factors.^[^ ^8^, ^47^, ^48^ ^]^ For the research, the study used only mature‐stage milk from days when the donor and infant were feeling healthy. The milk was consistently collected in the morning, after the infant's first feeding of the day. By using one donor, it was known that maternal health and environmental factors were consistent and unlikely to affect study outcomes. The used of one donor helped us eliminate many confounding variables that typically present in human research.

The sole human milk donor was recruited according to the Institutional Review Board (IRB) protocol number STUDY2019_00000084 at Carnegie Mellon University. The donor met the following inclusion criteria: donors must be over 18 years old, must be breastfeeding a child born in the past 24 months at the time of donation, and must be in general good health. For this study, all milk samples came from a single donor when the donor was between 3 and 8 months postpartum. Each milk donation was approximately 50 mL in volume, and all milk was processed for experiments immediately after expression and was not frozen.

Cells were isolated from fresh human milk using the method described above.^[^ ^19^ ^]^

### 2.5 Simulated Gastric Digestion

Simulated gastric digestion was performed as previously described^[^ ^21^ ^]^ and modified to mimic infant digestive conditions.^[^ ^17^, ^22^ ^]^ Freshly expressed whole breast milk was diluted 1:1 in a buffer consisting of 34 mM sodium chloride and 19 mM potassium chloride, then adjusted to pH 4 with 1 M hydrochloric acid (HCl). The solution was spiked with 80 U mL^−1^ porcine pepsin (Sigma) and incubated at 37 °C with gentle shaking for 30 min. The digestion reaction was quenched by 1:1 dilution into cold PBS (pH 7.4). Cells were isolated from the digestion solution by centrifugation at 800 × *g*, 4 °C for 20 min followed by two more washes in PBS to remove the milk fat and protein components. The cells were then resuspended in PBS or assay‐specific buffers as described below.

For the pH buffering experiment shown in Figure 3A, 1 M HCl was added to either the base simulated gastric fluid (SGF) solution, whole milk, or a 1:1 (v/v) mixture of whole milk and SGF. pH was measured with a pH meter.

### 2.6 Milk Cell Flow Cytometry

Several flow cytometry‐based assays were used to evaluate cell viability, mechanism of cell death, and cell phenotype. To assess cell viability and mechanism of death, an Annexin V‐FITC/PI assay (Cayman Chemical), CellEvent Caspase 3/7 activity assay (Thermo Fisher Scientific), and a 5,5',6,6' tetrachloro‐1,1',3,3'‐tetraethylbenzimi‐dazoylcarbocyanine iodide (JC‐1) mitochondrial membrane potential assay (Thermo Fisher Scientific) were used. All were performed according to the manufacturer's protocols with subsequent flow cytometry analysis using a NovoCyte 3000 (Agilent). To characterize the cell populations remaining after simulated gastric digestion, both live cell assays and antibody staining were used.

The study assessed the cells for stem cell‐like properties using an ALDEFLUOR assay according to the manufacturer's protocol (StemCell Technologies).^[^ ^23^ ^]^ High aldehyde dehydrogenase (ALDH )signal indicates stem‐like phenotypes. After staining with ALDEFLUOR at 37 °C for 30 min, cells were washed in PBS, counterstained with PI to exclude dead cells, and immediately analyzed by flow cytometry.

For experiments involving antibody staining, cells were stained for 30 min with LIVE/DEAD Fixable Yellow Dead Cell dye (Thermo Fisher Scientific) according to the manufacturer's protocol, and then fixed and permeabilized using a flow cytometry fixation and permeabilization buffer kit (R&D Systems). Cells were stained with antibodies at a 1:100 dilution (**Table** **1**), washed to remove unbound antibody, and subsequently analyzed by flow cytometry.

**Table 1:** Antibodies used for flow cytometry

Target	Cell type	Fluorophore	Manufacturer	Cat no.
EpCAM	Epithelial cells	PE‐Cy7	eBioscience	25‐9326‐42
CK18	Luminal epithelial cells	FITC	Invitrogen	MA110327
Vimentin	Mesenchymal cells	APC	Invitrogen	MA528601
CD45	Immune cells	AlexaFluor 488	R&D systems	FAB1430G
NANOG	Stem cells	PE	Invitrogen	PA546891
SOX2	Stem cells	eFluor 660	eBioscience	50‐9811‐82
TRA‐1‐60	Stem cells	FITC	Invitrogen	MA1023D488X
CD49f	Epithelial progenitors	eFluor 450	eBioscience	48‐0495‐82

### 2.7 qRT‐PCR

Following simulated gastric digestion, total RNA was isolated from milk cells using an Isolate II RNA Mini Kit (Bioline) according to the manufacturer's protocol. Paired samples of undigested milk were used as untreated controls. qRT‐PCR was performed using SensiFAST SYBR One‐Step master mix (Bioline) and a ViiA 7 Real‐Time PCR system (Applied Biosystems). Gene expression fold change following simulated digestion was determined using the ΔΔCt method^[^ ^24^ ^]^ in which genes of interest were normalized to the expression of *GAPDH* (**Table** **2**).

**Table 2:** Primers used for qPCR

Gene	Forward primer	Reverse primer
NANOG	AACCTCAGCTACAAACAGGTGA	TCTGCGTCACACCATTGCTA
SOX2	CCATGCAGGTTGACACCGTTG	TCGGCAGACTGATTCAAATAATACAG
OCT4	GACAGGGGGAGGGGAGGAGCTAGG	CTTCCCTCCAACCAGTTGCCCCAAAC
MDR1	GCTCAAGTTAAAGGGGCTAT	GCCAACCATAGATGAAGGAT
BCRP	TTCGGCTTGCAACAACTATG	TCCAGACACACCACGGATAA

### 2.8 Data Analysis and Statistics

Statistical analysis and graphical data presentation were performed in Prism 8 (GraphPad Software). Differences between two groups were analyzed using Student's *t*‐test, and differences between more than two groups were analyzed using a one‐way ANOVA with a post‐hoc Tukey test. Differences were considered statistically significant at *p* < 0.05. Data shown represent the mean ± standard deviation of three or more independent experimental replicates (breast milk collected on different days) as indicated. Flow cytometry data were analyzed using NovoExpress software (Agilent).

## 3 Results

The goal of this study was to establish the fate of maternal milk cells in the infant's gastrointestinal tract. Towards that end, we examined how gastric digestion (simulated and in mice) affects milk cell viability, the mechanism by which cell death occurs, and the influence of the non‐cellular milk components on those processes.

### 3.1 Milk Cells Must Survive Digestion to Reach the Intestine

**Figure 1:**
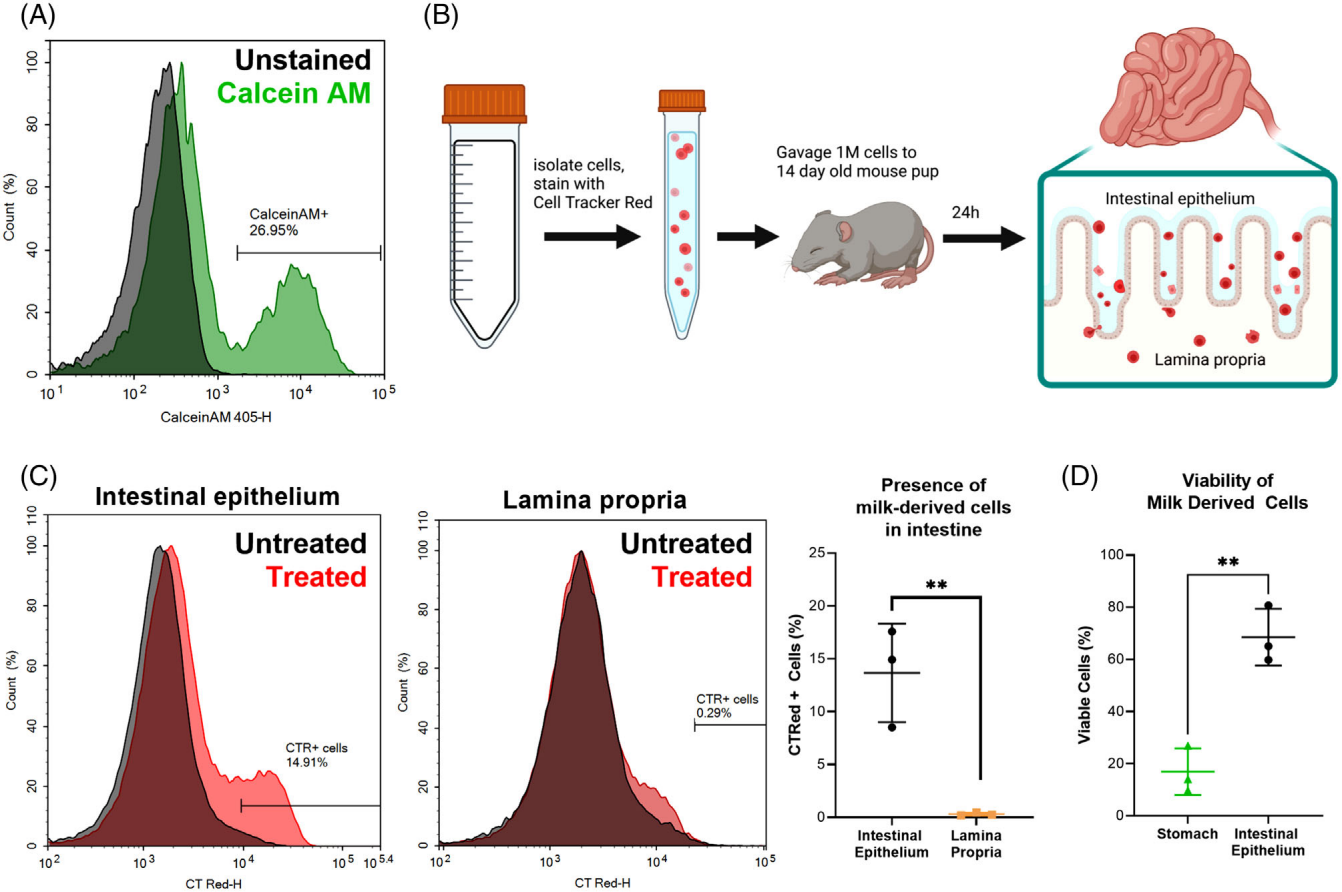
A fraction of milk‐derived cells survives infant gastric digestion and reaches the intestine. A) Milk cells were isolated from the stomachs of 14‐day‐old mouse pups, stained for viability with calcein AM, and analyzed by flow cytometry. The cells in the milk bolus exhibited ≈27% viability (green histogram) compared to the unstained control (gray histogram). B) Schematic showing the experimental method to identify milk‐derived cells in the intestines of mouse pups. Human milk cells were stained with CellTracker Red (CTR), washed to remove excess dye, and delivered to 14‐day‐old mouse pups by oral gavage. 24 hs later, the intestines were dissected and digested in collagenase to produce single cell suspensions from both the intestinal epithelium and lamina propria. C) Flow cytometry histograms show the presence of CellTracker Red‐stained milk cells in the mouse intestine. After dissociating the epithelial cells from the intestine, the lamina propria was digested into a single cell suspension. the left histogram shows the epithelial layer of the intestine, and the right histogram shows the lamina propria; for each histogram, red represents a sample from a mouse pup given CTR‐stained milk cells, and gray represents a mouse pup given PBS. While ≈15% of cells in the intestinal epithelium were positive for CellTracker Red, a much smaller number of milk‐derived cells appeared in the lamina propria. *N* = 3 mice, **p < 0.01 by Student's *t*‐test. D) The viability of the milk‐derived cells in the stomach and intestine was measured using propidium iodide analysis by flow cytometry. Although only ≈20% of the milk cells in the stomach were viable, a significantly higher number of those that reached the intestine were viable. *N* = 3 mice, **p < 0.01 by Student's *t*‐test. Error bars represent standard deviation.

First, we used a mouse model to quantify the number of milk cells that survive infant gastric digestion and reach the intestine in vivo. For these studies, we used 14‐day old C57BL/6 mouse pups because this is the age when pups are growing rapidly and beginning their transition from drinking milk to eating solid food. For comparison, human infants typically start to eat solid food around age 4–6 months; therefore, we consider 14 day old mouse pups to be roughly equivalent to a 4‐month‐old human infant.^[^ ^25^, ^26^ ^]^ We expected that most of the milk‐derived cells would die due to either stomach acid or the digestive enzymes that break down biomacromolecules and fatty acids. Analysis by flow cytometry revealed that cells from the milk bolus isolated from the stomachs of mouse pups were ≈20–30% viable (**Figure** **1** **A,D**). This is not surprising because, while the gastric environment of mouse pups and human infants is substantially less harsh than that of adults (pH: infant ≈4, adult ≈1–2; pepsin: infant ≈268 U mL^−1^, adult ≈2000 U mL^−1^),^[^ ^22^, ^27^ ^]^ it is nonetheless sufficient to digest biomacromolecules.

Next, we determined if live milk cells could be detected in the intestines of 14‐day old mouse pups. We used CellTracker Deep Red to label human milk cells and orally delivered labeled cells to mouse pups (Figure 1B). We found labeled cells in the intestinal epithelium (Figure 1C). Moreover, the labeled cells in the intestine were nearly 100% viable, which is significantly greater than the viable fraction of cells within the stomach (Figure 1D). Gating schemes for intestinal flow cytometry are shown in Figure S1, Supporting Information. These data demonstrate that most ingested mouse milk cells do not survive gastric digestion, but the labeled cells that are retained in the intestines are nearly all viable.

### 3.2 Breast Milk Cells Apoptose during Digestion Due to Low pH and Pepsin

Given that most milk cells die during the digestive process, we next probed the mechanism of cell death. Throughout the remainder of this study, we analyzed human milk, and modeled the infant digestion process in vitro using SGF with a pH of 4.^[^ ^28^ ^]^ Freshly expressed human milk was diluted 1:1 in infant SGF at pH 4 supplemented with pepsin, and then incubated at 37 °C with gentle shaking for 30 min. Following digestion, cells were isolated from the milk by centrifugation and resuspended in PBS for viability staining and analysis.

**Figure 2:**
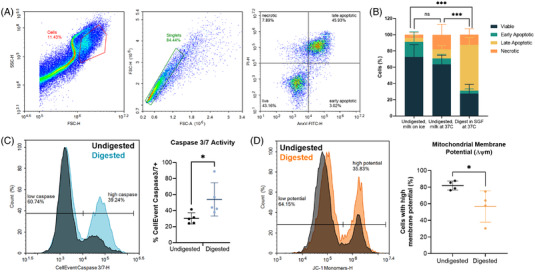
Human milk cells undergo classical caspase‐mediated apoptosis during simulated gastric digestion. A) Representative flow cytometry scatter plots show cells isolated from a sample of human milk, and the populations of viable and nonviable cells as identified by staining with Annexin V and propidium iodide. Propodium iodide on the *y*‐axis and Annexin V on the *x*‐axis identified viable, apoptotic, and necrotic cell populations. B) Human milk cells subjected to simulated digestion with simulated gastric fluid (SGF) for 30 min were less viable and more apoptotic than undigested cells as evidenced by AnnexinV/propidium iodide (PI) staining and flow cytometry analysis. Two undigested control populations were examined: milk cells immediately after isolation and those incubated at 37 °C in PBS for 30 min. ***p < 0.005 for viable fraction by Student's *t*‐test. C) Using the CellEvent Caspase 3/7 assay, digested breast milk cells exhibited increased caspase 3/7 activity, indicative of apoptosis, compared to undigested control. Flow cytometry histograms show digested cells (blue) are 54% apoptotic, and undigested cells (gray) are 30% apoptotic. *p < 0.05 by Student's *t*‐test. D) Using the JC‐1 assay, digested breast milk cells exhibited a lower fraction (reduced from ≈80% to ≈60%) of cells with high mitochondrial membrane potential, which indicates healthy mitochondria. *p < 0.05 by Student's *t*‐test. For all panels, *N* = 5, where each replicate was milk expressed on different days, and error bars represent standard deviation.

We hypothesized that digested breast milk cells undergo apoptotic or necrotic cell death. Apoptosis is a programmed cell death mechanism in which internal or external cues initiate a caspase‐mediated intracellular signaling cascade, resulting in cell death.^[^ ^29^ ^]^ In contrast, necrosis is generally a caspase‐independent process that occurs due to an overwhelming external chemical or physical injury.^[^ ^30^ ^]^ To interrogate this, we used an Annexin V/PI assay and a CellEvent Caspase Activation assay with subsequent analysis by flow cytometry (**Figure** **2**A). Cells undergoing apoptosis express the “eat me” signal, phosphatidylserine, on the outer leaflet of the cell membrane, which is detected by positive Annexin V staining. Necrotic cells stain negative for Annexin V, and positive for, which is a live cell‐impermeant DNA stain.^[^ ^31^ ^]^ These data revealed that simulated gastric digestion induced a ≈50% decrease in cell viability and that most cells died by apoptosis rather than necrosis (Figure 2B). To further support this, we used a CellEvent Caspase Activation assay to measure the activity of caspases 3 and 7, which increase during apoptotic cell death.^[^ ^31^ ^]^ We observed a significant increase in caspase activity in cells subjected to simulated gastric digestion (Figure 2C), indicating that caspase activation, and therefore apoptosis, is the major pathway for milk cell death during gastric digestion.

There are two main pathways by which cells undergo caspase‐mediated apoptosis: the intrinsic pathway initiated when an intracellular injury occurs, such as DNA damage or reactive oxygen production, and the extrinsic pathway initiated by extracellular cues. Extrinsic apoptosis is facilitated by ligand binding to cell surface death receptors or by cytotoxic stress. Cytotoxic stress then causes mitochondrial membrane permeabilization, which can be measured as a function of mitochondrial membrane potential.^[^ ^32^ ^]^ Healthy mitochondria exhibit high membrane potential (Δψm), while cells undergoing mitochondrial apoptosis exhibit mitochondrial depolarization and a decrease in membrane potential. This can be detected using a JC‐1 assay.^[^ ^33^ ^]^ The fluorescent JC‐1 probe is green‐fluorescent in healthy mitochondria, but undergoes a red shift during mitochondrial depolarization. Following simulated gastric digestion, we observed a 2‐fold increase in dying cells with low Δψm and a corresponding decrease in healthy cells with high Δψm (Figure 2D), suggesting that digested breast milk cells undergo apoptosis via the extrinsic mitochondrial pathway.

These studies provide new insight into how human milk cells respond to digestion. While previous research shows that a fraction of breast milk cells survive digestion to reach the intestines of breastfeeding infants, no studies have characterized that fraction of surviving cells, or the environmental cues which determine cell fate during gastric digestion.

### 3.3 Non‐Cellular Milk Components Offer Mild Protection from Digestion

**Figure 3:**
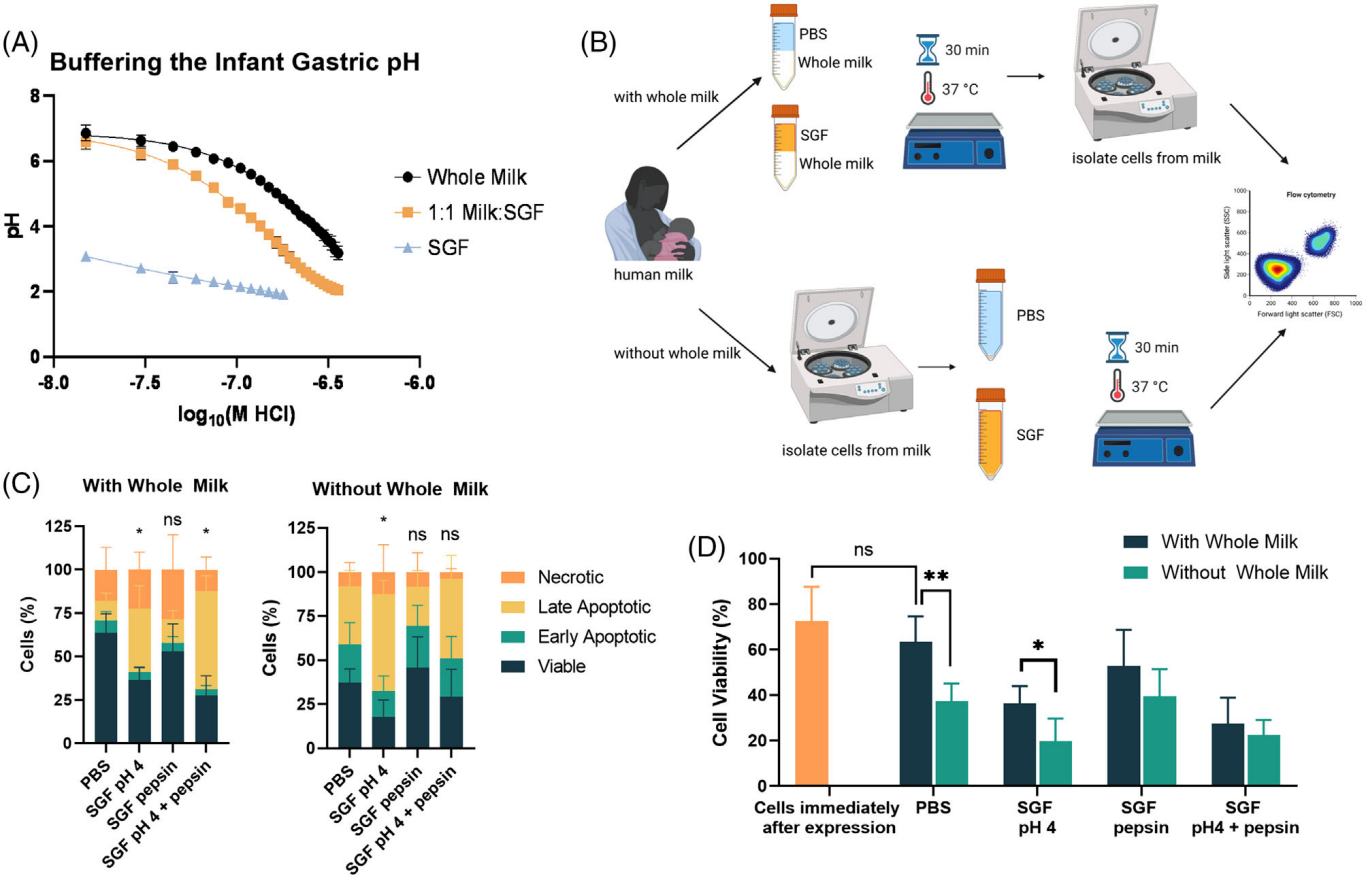
The non‐cellular components of milk buffer the pH of the gastric environment and provide mild protection against cell death from stomach acid. A) Milk buffered the acidic pH of the stomach environment. In this experiment, pH was tracked as hydrochloric acid (HCl) was added to whole milk (black circles), simulated gastric fluid (SGF, blue triangles), or milk and SGF at a 1:1 v/v ratio (mustard squares). *N* = 3 replicates, with error bars representing standard deviation. B) To determine the role of the non‐cellular components of milk in protecting cells in the stomach, human milk cells were subjected to gastric digestion under two conditions. For digestion with the non‐cellular milk components, equal volumes of whole milk and SGF were combined and incubated for 30 min. For digestion without the milk components, cells from the milk were first isolated by centrifugation, and then resuspended in SGF. For both conditions, PBS was used as a control for SGF. Following simulated digestion, cells were stained with Annexin V and propidium iodide and analyzed for viability by flow cytometry. C) For both treatments, acidic pH was the greatest contributor to cell death in the stomach. Data shown is from *n* = 5 replicates from milk expressed on different days. *p < 0.05 for viable fraction compared to PBS control. Error bars represent standard deviation. D) The non‐cellular components of milk reduce cell death, both in PBS and in the acidic environment of the stomach. For comparison, the viability of cells immediately after milk expression, without any incubation period, is shown at left. While the non‐cellular components of milk can protect from cell death due to low pH, they do not offer significant protection against the combination of low pH and pepsin. Data shown is from *n* = 5 replicates from milk expressed on different days. *p < 0.05, **p < 0.01 by Student's *t*‐test. Error bars represent standard deviation.

Milk is a complex fluid that contains bioactive macro‐ and small molecules in addition to the cells. We asked whether the non‐cellular components of whole milk might protect the cells from death in the stomach environment by buffering the gastric pH. To examine this, we titrated either SGF, human milk, or a 1:1 (v/v) mixture of SGF and milk with hydrochloric acid and measured the resulting pH. We found that whole human milk buffered the pH even when it was diluted 1:1 with SGF (**Figure** **3**A).

To determine whether this buffering effect can protect milk cells in the stomach, we measured the viability of milk cells after simulated gastric digestion with either whole milk in the SGF solution or the cells isolated from milk re‐suspended into SGF. A schematic of this process is shown in Figure 3B. We then compared viability for these two cases to understand the protective role of non‐cellular milk components.

Additionally, we were interested in identifying which digestive components are responsible for triggering milk cell apoptosis. Therefore, we subjected freshly expressed breast milk to “deconstructed” simulated gastric digestion. Here, the cells were diluted in one of three types of SGF: 1) no pepsin, pH 4; 2) with pepsin, pH 7; or 3) with pepsin, pH 4. The negative control samples were diluted in PBS. For each sample, milk cell viability was measured for the two cases described above: with and without other milk components present in the digestive environment. Annexin V/PI analysis showed that low pH contributes more to cell death than digestive enzymes. We observed significant drops in viability for cells incubated at pH 4 with or without pepsin, while the addition of pepsin did not produce an additional significant decrease in viability (Figure 3C). Pepsin without acid was insufficient to reduce cell viability, likely because pepsin activity is highest at pH 1.5–2.5 and is inactive at pH 6.5 and above.^[^ ^34^ ^]^ Because the pH of milk diluted in SGF is around 7.0, pepsin is mostly inactive in these samples.

After comparing the effects of the different components of digestive fluid, we studied the effects of the non‐cellular milk components on cell survival. In Figure 3D, we have re‐plotted the viable fraction of cells from Figure 3C, in order to more directly compare the survival of cells with and without whole milk components. We found that more of the cells without the protection of the milk died from low pH and incubation at 37 °C. There were no significant viability differences between the cells with and without milk components for the samples digested with both acidic pH and pepsin (Figure 3D). This suggests that milk provides only mild protection from digestion and cannot protect against the combination of an acidic environment and enzymes.

### 3.4 Milk‐Derived Immune Cells Are Most Likely to Die during Gastric Digestion

Milk contains many cell types that have historically not been well characterized.

**Figure 4:**
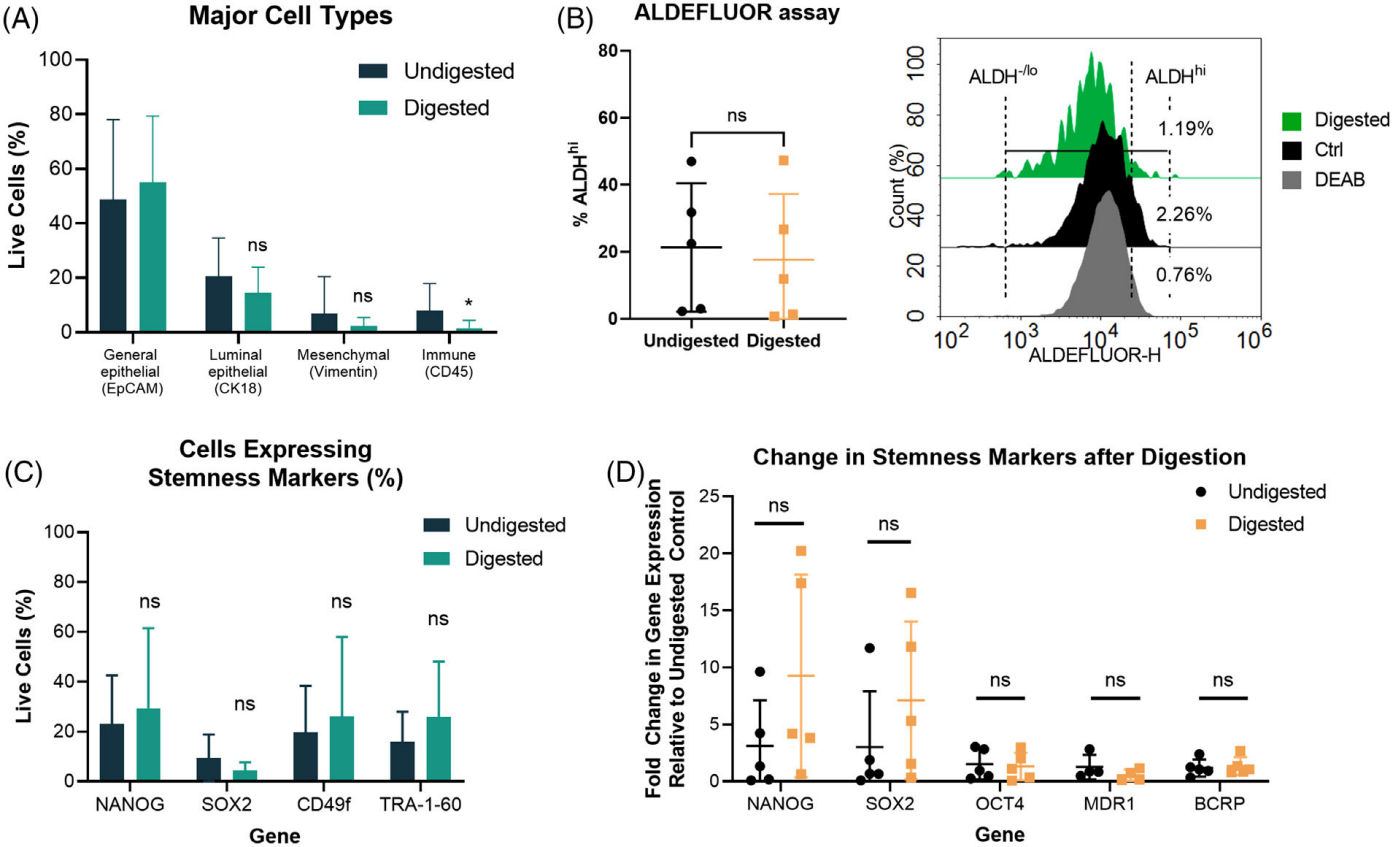
Infant gastric digestion kills all cell types equally, except for immune cells, which are less resilient to digestion. A) Cell populations were identified using antibody staining and subsequent flow cytometry analysis. The relative percentages of defined viable cell populations in milk before and after digestion did not change, with the exception of immune cells, which did not survive digestion. (*n* = 6 replicates from milk expressed on different days). B) The ALDEFLUOR assay measured stem cell populations before and after simulated digestion. High ALDH signal indicates stem‐like phenotypes. ALDEFLUOR staining found no change in stemness following digestion. DEAB is an inhibitor of ALDH and was used to control for background fluorescence (*n* = 5). C) Digested and undigested milk cells were analyzed by flow cytometry for the presence of stem and progenitor cell markers NANOG, SOX2, CD49f, and TRA‐1‐60. No changes in stemness were detected following digestion (*n* = 5). D) Digested and undigested milk cells were analyzed by qPCR for the presence of stem cell markers. No significant changes were detected (*n* = 5). In all panels, significance was determined by Student's *t* test compared to undigested control cells, with *p < 0.05. Error bars represent standard deviation.

We characterized the proportions of different cell populations in milk by flow cytometry, using EpCAM, CK18, Vimentin, and CD45 to identify populations of epithelial cells, luminal epithelial cells (lactocytes), mesenchymal cells, and immune cells, respectively (**Figure** **4**A). We found that overall, gastric digestion did not select for a specific resilient cell type, and all the cell types were killed in roughly equal proportions. The only exception was immune cells, of which almost all died during gastric digestion. Flow cytometry gating schemes are shown in Figures S2,3S, Supporting Information. However, a limitation of our study was that we did not use a nuclear stain for flow cytometry.^[^ ^39^, ^40^ ^]^ It is possible that we have may have inadvertently characterized some milk fat globules or other membrane‐enclosed structures as cells.

Following the general characterization of cell populations in milk, we investigated stem cell populations. We first used the ALDEFLUOR assay to identify cells with stem‐like phenotypes. Stem and progenitor cells have higher levels of aldehyde dehydrogenase (ALDH) activity, and when incubated in a buffer containing ALDH substrate, they convert the substrate into a fluorescent molecule that can be detected by flow cytometry.^[^ ^41^ ^]^ Diethylaminobenzaldehyde (DEAB) is an inhibitor of ALDH and was used as a negative control. We found that simulated digestion did not change the percentage of cells with high ALDH activity (Figure 4B), and in both the digested and undigested samples, the overall levels of ALDH^hi^ cells were low, around 2% of live cells.

We also tested for stem cell markers at the RNA and protein levels. Using flow cytometry, we analyzed digested and undigested milk cells looking for the stem and progenitor cell markers NANOG, SOX2, CD49f, and TRA‐1‐60 (Figure 4C). In all cases, there was no change in the proportion of cells expressing these markers following simulated digestion. Finally, we performed qPCR looking for the markers *NANOG*, *SOX2*, *OCT4*, *MDR1*, and *BCRP* (Figure 4D). *NANOG*, *SOX2*, and *OCT4* are transcription factors associated with pluripotent stem cells;^[^ ^4^ ^]^ MDR1 and BCRP are efflux transporters that tend to be highly expressed in cells with stem‐like phenotypes.^[^ ^42^ ^]^ qPCR analysis at the RNA level, like flow cytometry analysis at the protein level, showed no change in the expression of these genes. Together, these data suggest that the relative percentage of stem‐like cells does not change in the living cell population before and after digestion. In other words, digestion neither selectively kills stem cells nor selectively allows stem cells to survive.

## 4 Discussion

### 4.1 Possible Explanations for Cell Survival in the Mouse Model

In our study of milk cells delivered to mice, we saw that few cells survived digestion in the stomach. However, out of the portion of cells in the intestine that we observed containing the red cell tracker dye, the majority were viable. We believe there could be three reasons for these observations: 1) most milk cells die during digestion, with only a small fraction surviving and integrating into intestinal tissue; 2) milk cells shed exosomes and apoptotic bodies containing the fluorescent dye that are uptaken by live cells in the intestinal epithelium; and/or 3) dead or dying milk cells are taken up by phagocytic cells in the intestinal epithelium. We doubt that fluorescent signal could be due to free CellTracker dye, because the dye fluoresces only when cleaved by intracellular esterases and then binds covalently to proteins inside the cell.^[^ ^43^, ^44^ ^]^ Although the possibility of exosomes and/or phagocytotic cells containing CellTracker is an interesting topic for future investigation, in this study we have focused on the populations of cells in milk and their survival.

### 4.2 The Majority of Cell Death Occurs by Caspase‐Mediated Apoptosis

The infant digestive conditions are mild compared to adult conditions—overall, infants have a higher gastric pH, and lower levels of digestive enzymes. The literature often reports seemingly contradictory values for infant gastric pH, partly because pH varies depending on gestational age, fed versus fasting state, and formula feeding versus breastfeeding. Infants are born with a neutral stomach pH, and undergo rapid developmental changes in the first days and weeks of life.^[^ ^28^ ^]^ Reported values for infant stomach pH range from 1.6 to 4.6 for fasted infants, and a pH of around 6 for fed infants.^[^ ^45^ ^]^ For comparison, the typically cited value for adult fasted gastric pH is 1.7.^[^ ^46^ ^]^ We chose to model the human infant digestion process in vitro using SGF with a pH of 4.^[^ ^28^ ^]^

Since the stomach environment is acidic, we expected that necrosis would be the primary mechanism of cell death under simulated digestion, and we were surprised by our results. However, the finding that breast milk cells undergo caspase‐mediated, mitochondrial apoptosis may enable intervention with the apoptosis pathway. This could increase the fraction of cells that survive digestion and enable the use of cells for neonatal intestinal therapy or oral drug delivery.

### 4.3 Milk Proteins and Fats Offer Mild Protection from Digestion

In this study, we were interested in whether the liquid matrix of whole milk helps cells survive digestion. The major protein constituents of human milk include α‐lactalbumin, lactoferrin, caseins, IgA, lysozyme, and albumin.^[^ ^49^ ^]^ Human milk fat, though highly variable based on maternal diet and infant feeding schedule, contains large quantities of palmitic and oleic acids. While much is known about the nutritional benefits these components offer to infants, nothing is known about how they impact milk cell viability during digestion. Understanding how specific protein and fat components interact with milk cells would be an interesting topic for future study.

### 4.4 Live Immune Cells in Milk Are Not Necessary to Offer Immune System Benefits to Infants

The majority of the cells in milk are derived from the mammary epithelium, including lactocytes and mammary progenitor cells.^[^ ^5^, ^35^, ^36^ ^]^ Immune cells make up a relatively small proportion of cells in milk in a healthy mother–infant dyad, but much of the previously published research on milk has been focused on the role of the immune cells on the infant's developing immune system.^[^ ^37^ ^]^ Stem cells have also been difficult to characterize and there is considerable debate on how many of the cells in milk can truly be considered stem cells.^[^ ^38^ ^]^

Our research shows that most immune cells die during digestion. However, the immune benefits of breastfeeding have been well‐studied. It is well known that breast milk contains high numbers of antibodies and proteins which can both offer temporary protection from infection while the infant's immune system develops, and educate the infant's immune system to prepare for more long‐term protection.^[^ ^50^, ^51^ ^]^ It has long been hypothesized that live immune cells from milk have an important role to play in the infant intestine and beyond.^[^ ^13^ ^]^ At the same time, others hypothesize that the role of immune cells in milk is to protect the mother. For example, immune cell populations have been shown to increase when mother or infant has an illness or infection.^[^ ^8^, ^48^ ^]^ Taken together, we can consider milk as part of a dynamic system that responds to both the needs of the infant and the mother.^[^ ^52^ ^]^

We hypothesize that infants do not require live maternal immune cells to reach the intestine to gain immune benefits from milk. Instead, the immune benefits come from antibodies, proteins, and commensal bacteria that more easily survive digestion, freezing, and storage. This finding may suggest that infants who consume frozen breast milk or donor milk will still receive the immune benefits of breastfeeding.

### 4.5 Future Research Directions

While other studies have examined the fate of proteins, fats, and miRNAs from milk during digestion, no study to date has examined the fate and survival of cells. Here, we showed that most maternal milk cells do not survive digestion and that they die via apoptosis. Although the milk matrix, i.e., the non‐cellular components of milk, buffers the acidic pH of the stomach, it offers minimal protection from gastric digestion. Previous reports have shown cell‐specific effects of milk‐derived cells in the intestine^[^ ^13^ ^]^ or shown that maternal immune cells in milk educate the infant immune system.^[^ ^53^ ^]^ However, since only a minority of cells survive infant gastric digestion, it is important to contextualize the reported effects of live cells, and understand that they are only a small part of a vast ecosystem. The data suggest that future research should focus less on mechanisms requiring large numbers of healthy milk cells to reach the intestine, and instead should examine other explanations for the apparent phenomenon of maternal cells in the infant's intestine and systemic organs. For example, the role of maternal exosomes and/or dead cells in the infant's intestinal epithelium deserves more attention. Milk is complex and has many bioactive components, and understanding their fate in the infant's intestine is more nuanced than previously described in the literature. We anticipate that the results presented in this study will catalyze those efforts.

## Conflict of Interest

The authors declare no conflict of interest.

## Author Contributions

All authors conceived the experiments and wrote the manuscript. J.R.M. and R.D. conducted the experiments, analyzed the results, and wrote the manuscript. All authors revised it and approved the final version of the manuscript.

## Supporting information

Supporting information.

## Data Availability

The data that supports the findings of this study are available in the supplementary material of this article.
