# Supplementary material for: The Effect of Infant Gastric Digestion on Human Maternal Milk Cells
Source: Mol Nutr Food Res. 2022 Aug 31;66(19):2200090. doi: 10.1002/mnfr.202200090 (PMC9532377; doi:10.1002/mnfr.202200090)
Supplement: Supplementary file 1 — Supporting information. [file MNFR-66-2200090-s001.pdf]

The effect of infant gastric digestion on human maternal milk cells

Supplementary Information

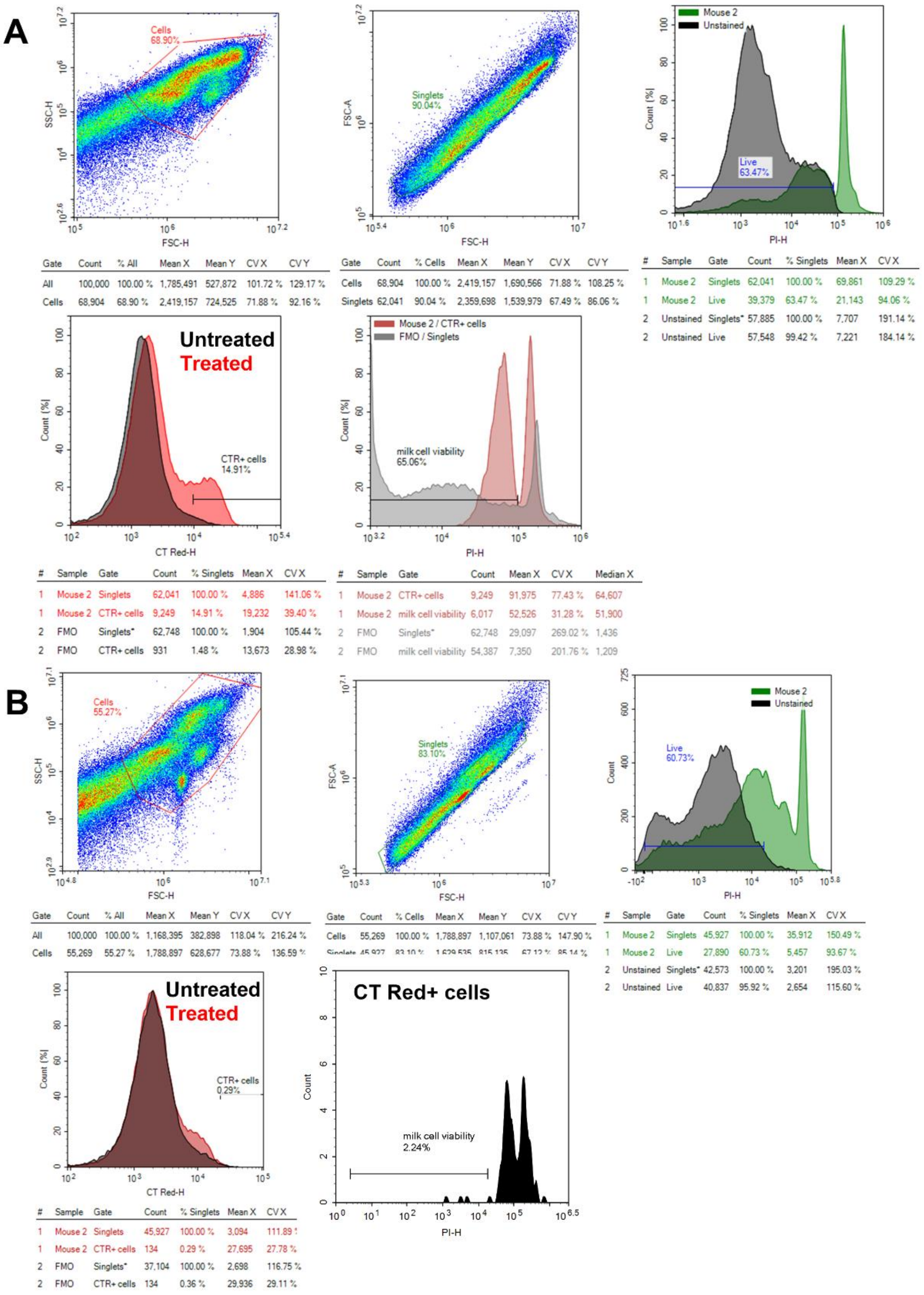

Figure S1. Representative flow gating strategy for mouse intestine samples.

A) Gating for intestinal epithelium.
B) Gating for lamina propria.

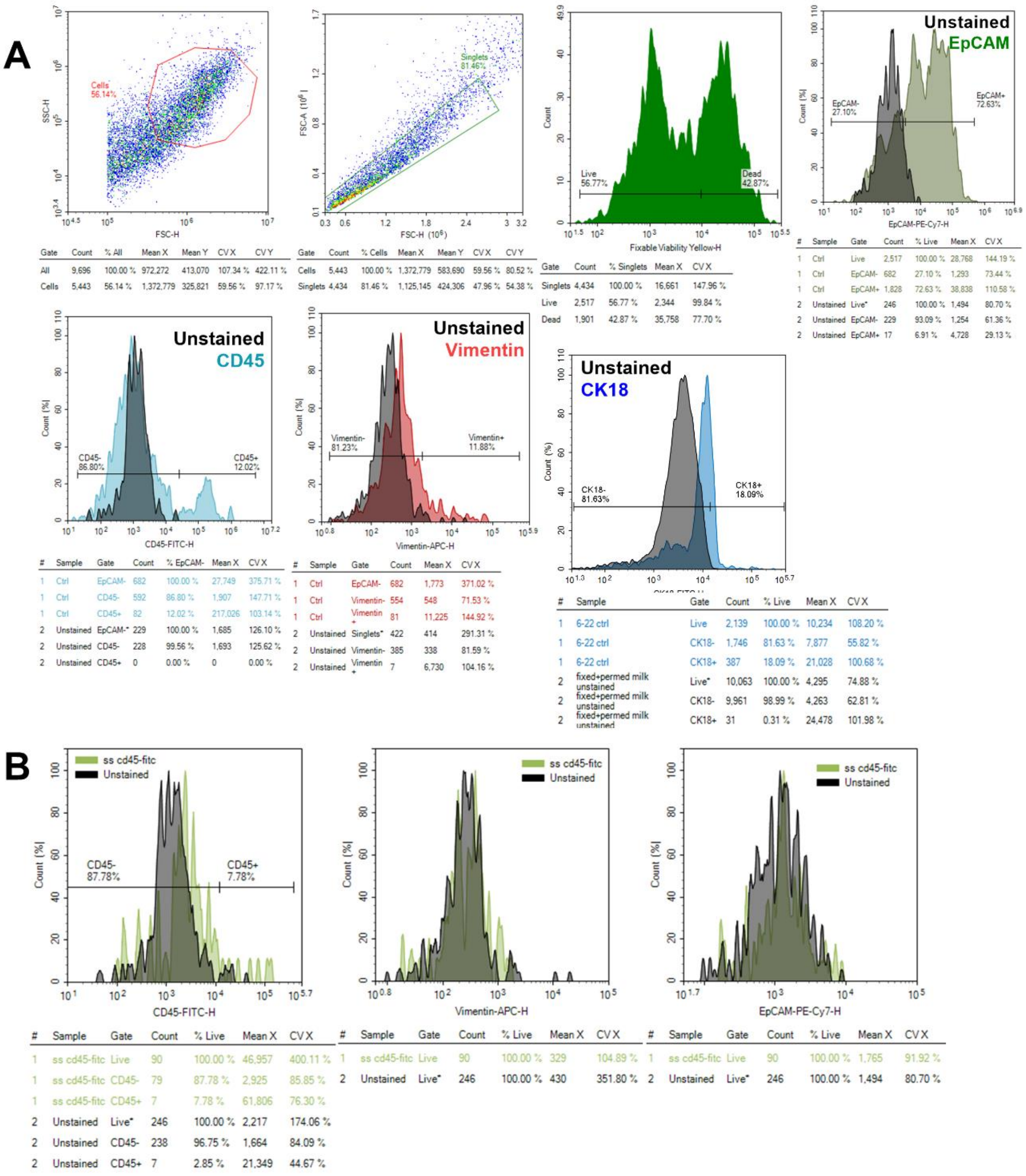

Figure S2. Representative flow gating and cell counts for the major cell populations in milk.

A) Representative plots showing the flow gating scheme to identify the major populations of cells in human milk.
B) Representative plots showing the single stained control for CD45-FITC. The staining does not bleed over into the channels for the other cell types.

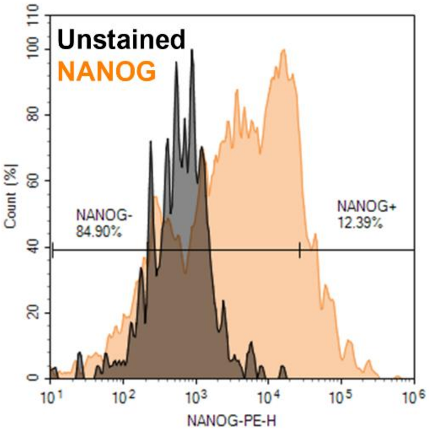

#	Sample	Gate	Count	% Live	Mean X	CV X
1	Ctrl	Live	2,801	100.00 %	13,202	215.10 %
1	Ctrl	NANOG-	2,378	84.90 %	6,238	112.35 %
1	Ctrl	NANOG+	347	12.39 %	63,836	89.24 %
2	Unstained	Live*	246	100.00 %	946	148.30 %
2	Unstained	NANOG-	242	98.37 %	962	146.29 %
2	Unstained	NANOG+	0	0.00 %	0	0.00 %

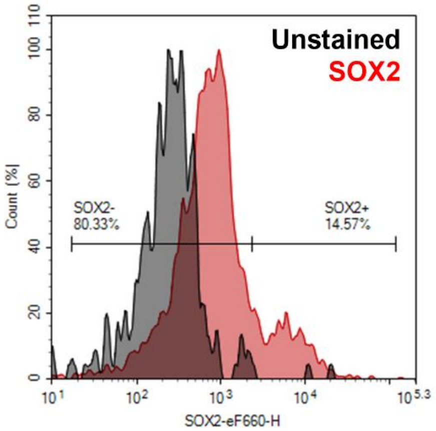

#	Sample	Gate	Count	% Live	Mean X	CV X
1	Ctrl	Live	2,801	100.00 %	30,782	1,718.14 %
1	Ctrl	SOX2-	2,250	80.33 %	769	62.10 %
1	Ctrl	SOX2+	408	14.57 %	7,604	89.99 %
2	Unstained	Live*	246	100.00 %	430	351.80 %
2	Unstained	SOX2-	224	91.06 %	323	95.17 %
2	Unstained	SOX2+	3	1.22 %	11,466	79.76 %

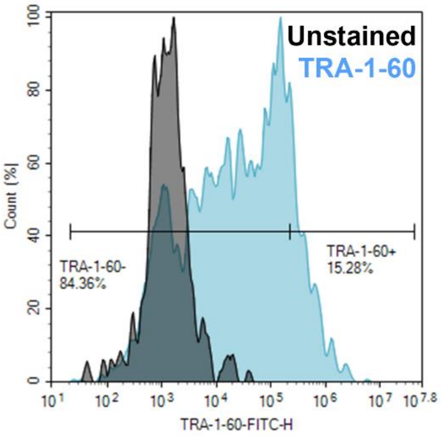

#	Sample	Gate	Count	% Live	Mean X	CV X
1	Ctrl	Live	2,801	100.00 %	124,127	228.91 %
1	Ctrl	TRA-1-60-	2,363	84.36 %	45,505	127.02 %
1	Ctrl	TRA-1-60+	428	15.28 %	561,105	95.14 %
2	Unstained	Live*	246	100.00 %	2,217	174.06 %
2	Unstained	TRA-1-60-	245	99.59 %	2,227	173.57 %
2	Unstained	TRA-1-60+	0	0.00 %	0	0.00 %

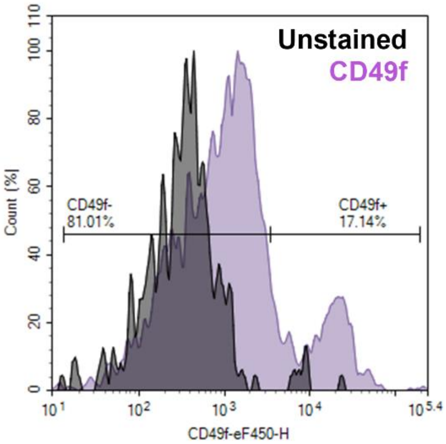

#	Sample	Gate	Count	% Live	Mean X	CV X
1	Ctrl	Live	2,801	100.00 %	5,179	417.63 %
1	Ctrl	CD49f-	2,269	81.01 %	1,058	77.99 %
1	Ctrl	CD49f+	480	17.14 %	17,365	89.07 %
2	Unstained	Live*	246	100.00 %	675	285.12 %
2	Unstained	CD49f-	227	92.28 %	414	79.93 %
2	Unstained	CD49f+	7	2.85 %	10,325	57.21 %

Figure S3. Representative flow gating and cell counts for the stem cell markers in milk.
